# Current Effective Therapeutics in Management of COVID-19

**DOI:** 10.3390/jcm11133838

**Published:** 2022-07-01

**Authors:** Kavya Atluri, Iris Aimlin, Shitij Arora

**Affiliations:** 1 Department of Bioinformatics, University of California, Los Angeles, CA 90095, USA; katluri12@gmail.com; 2 Department of Internal Medicine, Montefiore Medical Center, New York, NY 10467, USA; ilin@montefiore.org; 3 Department of Internal Medicine, Division of Hospital Medicine, Montefiore Medical Center, Albert Einstein College of Medicine, New York, NY 10461, USA

**Keywords:** COVID-19, therapeutics, omicron, coronavirus disease 2019, monoclonal antibodies, Casirivimab plus imdevimab, Sotrovimab, bebtelovimab, remdesivir, molnupiravir, paxlovid, evusheld, corticosteroids, baricitinib, tocilizumab, Anakinra, anticoagulation, timeline

## Abstract

The current pandemic due to the SARS-CoV-2 virus has caused irreparable damage globally. High importance is placed on defining current therapeutics for Coronavirus Disease 2019 (COVID-19). In this review, we discuss the evidence from pivotal trials that led to the approval of effective therapeutics in the treatment and prevention of COVID-19. We categorize them as effective outpatient and inpatient management strategies The review also attempts to contextualize the efficacy of therapeutics to the emerging variants. Vaccines, which remain the most effective prevention against hospitalization and deaths is not included in this review.

## 1. Introduction

Identifying effective therapeutic strategies for Coronavirus Disease 2019 (COVID-19) in a timely manner has been one of the most significant challenges. As of 15 June 2022, there is an excess of 85 million cases and 1 million deaths in the United States [1], as well as over 534 million confirmed cases and 6.3 million deaths globally [2]. At the time of writing this paper, we have at our disposal several effective therapeutics that may be used based on the timing of patient presentation and disease severity. Additional considerations for the treatment prioritization and management of high-risk patients include old age, high body mass index (BMI) and underlying comorbidities, including but not limited to diabetes, hypertension, obesity and chronic lung and heart diseases [3,4]. While there is a constantly changing landscape with the emergence of new variants, we discuss in this brief review the therapeutics that have shown efficacy in the pivotal trials.

1. Outpatient management: monoclonal antibodies, nirmatrelvir [PF-07321332] and ritonavir (Paxlovid), molnupiravir, remdesivir (Veklury) and bebtelovimab.
2. Inpatient management: remdesivir, corticosteroids, tocilizumab, baricitinib and anakinra.

Vaccines remain the most effective preventive strategy and a discussion is beyond the scope of this brief review. Heavily explored treatment options, such as hydroxychloroquine and Ivermectin, previously presented results indicating effectiveness against COVID-19 [5,6]. However, both were proven to exhibit no significant reduction in mortality [7]. Great financial expenditures and human risk were taken in trials for hydroxychloroquine and ivermectin to present no significant findings, and even increased mortality rates [7,8]. Preventative and cost-effective measures in the US remain in support of masks [9]. Additional effective measures include increased physical distancing [10] and ventilation of closed spaces [11].

## 2. Outpatient Management

### 2.1. Monoclonal Antibodies

Two monoclonal antibody regimens including (a) Casirivimab plus imdevimab (C+I) and (b) Sotrovimab (S) were approved for use in non-hospitalized patients with mild to moderate COVID-19 prior to the current Omicron surge [11]. C+I bind to the spike protein epitope, preventing attachment to the ACE 2 receptor [12]. Sotrovimab is a recombinant human IgG1-kappa mAb that also binds to an epitope on the spike protein receptor binding domain [12]; however, it does not compete with ACE-2 binding and likely inhibits an undefined step of viral replication at a later stage [13]. Both were approved for use in patients with the Delta variant who have risk factors for progression to severe disease (Table 1).

A number of questions remain unanswered and require further research. One of the key questions is the subsequent effect on vaccine-induced immune responses following monoclonal antibody treatment. Additionally, given the heterogeneity in patients who progress to severe disease, it may be possible to have a more precision-medicine-like approach in identifying the patients at the highest risk for progression. With the emergence of new variants, the efficacy of monoclonal antibodies would remain to be studied. Both C+I and S are effective for use against the Delta variant, and are approved for use with Delta. Though Sotrovimab was shown to significantly benefit patients across all Omicron subgroups compared to C+I in a recent study [15], due to changes in the binding site of the Omicron variant, they are not recommended for use in Omicron and BA.2 variants [16,17,18].

### 2.2. Bebtelovimab

Bebtelovimab is a new monoclonal antibody (mAb) to be used in patients with mild/moderate COVID-19 disease severity. As with C+I and S, bebtelovimab is a recombinant neutralizing mAb that also binds to the spike protein of SARS-CoV-2, but with increased efficacy for newer COVID-19 variants compared to the previous mAbs [19,20]. The NIH is advising that bebtelovimab be injected at 175 mg as a single IV injection, administered over 30 s in patients who are high-risk but non-hospitalized [11]. According to the BLAZE-4, a randomized phase 2 trial clinical trial that studied viral clearance in patients with mild to moderate COVID-19 at risk for progression, showed that the drug remains effective against the virus, but there are limited clinical efficacy data available. Currently, bebtelovimab is effective in vitro against all Omicron subgroups [11]. The BLAZE-4 trials began enrollment on 17 June 2020 and concluded the study on 20 October 2021 [21]. The FDA issued its emergency use authorization (EUA) on 11 February 2022 [22]. However, since there are no clinical efficacy data from placebo-controlled trials that evaluated the use of bebtelovimab in patients who are at high risk of progressing to severe COVID-19, the NIH recommends its use only in patients at risk of progression to severe COVID-19 for whom all other options are unavailable [11]. Bebtelovimab is shown to be effective in vitro against the BA.1, BA.1.1 and BA.2 Omicron subvariants [22]. Its use is authorized in adults, aged 12 years or older, and pediatric patients. This EUA excludes bebtelovimab use in patients with severe COVID-19 or who require oxygen therapy [22].

### 2.3. Remdesivir

**Figure 1:**
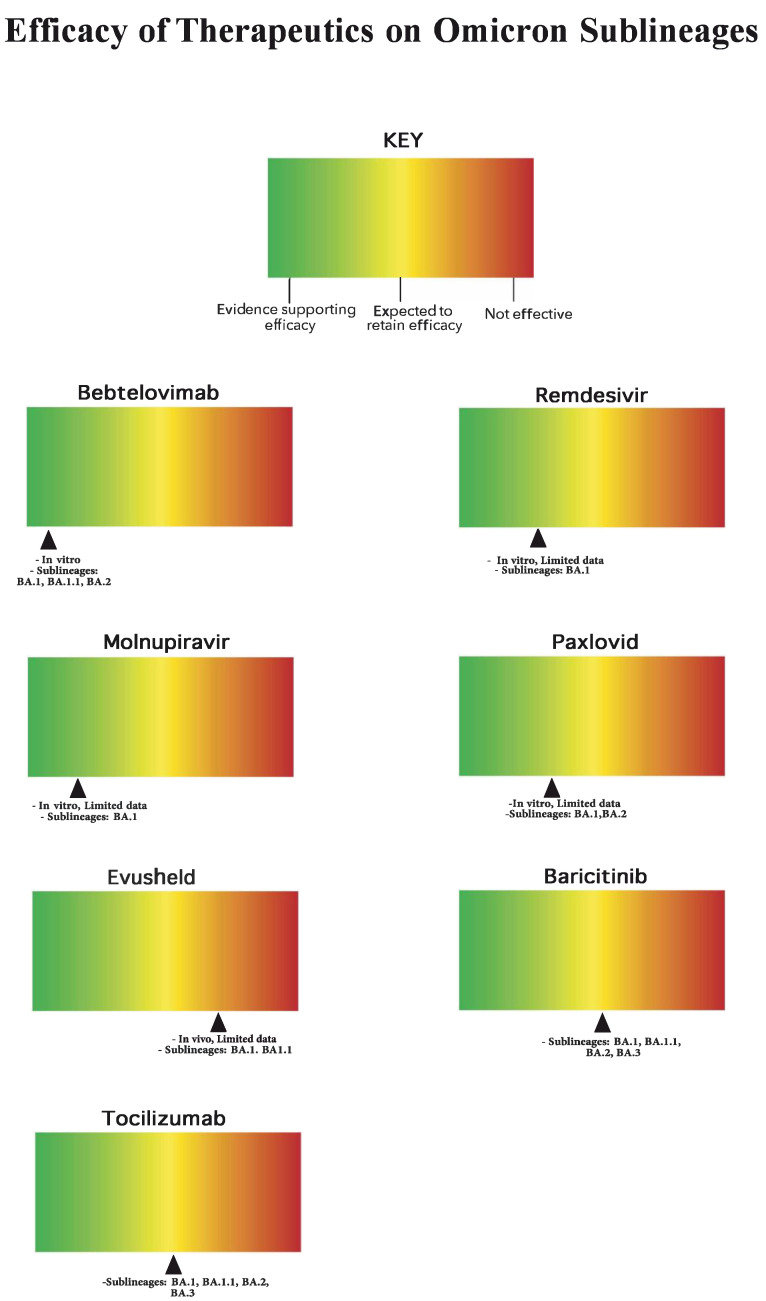
Therapeutic efficacies on Omicron sub-lineages.

Remdesivir is an antiviral treatment used in both hospitalized and non-hospitalized patients for mild/moderate and severe COVID-19 disease. Remdesivir prevents the RNA transcription of SARS-CoV-2 by binding to the viral RNA-dependent RNA polymerase, blocking viral replication [23]. The NIH advises 200 mg IV on Day 1 of symptom onset, along with 100 mg IV once daily on Days 2 and 3 in non-hospitalized patients, within the first 7 days of symptom onset [11]. According to the PINETREE trial, the number needed to treat to prevent hospitalization in non-hospitalized patients was 20 [24], and the hazard ratio (HR) was 0.13 with a 95% CI of 0.03–0.59 [25]. Remdesivir is the only drug that is FDA approved, securing approval on October 22nd, 2020. It is expected to be active in-vitro against the B.1.1.529 Omicron variant (Figure 1) [11,23,26]. However, there are limited in vivo data on remdesivir’s effects against Omicron [26].

### 2.4. Molnupiravir

Molnupiravir is an antiviral treatment for those with mild/moderate COVID-19 disease severity. The active form of molnupiravir is utilized as the substrate for viral RNA- dependent RNA polymerase instead of the coronavirus RNA genome. Therefore, replication of the COVID-19 genome is prevented and a mutated RNA is synthesized in its place [27]. The NIH recommends administering molnupiravir in non-hospitalized patients age 18 or older, 800 mg orally, twice daily for 5 days, only when paxlovid and remdesivir are unavailable [11]. In the MOVe-OUT trial, the number needed to prevent hospitalization for molnupiravir is 33; the treatment difference is −6.8% with a 95% CI= −11.3 to −2.4 [28]. The most interesting finding of the trial was the discrepancy between the interim results (48.2% efficacy) and the final results (29.9% efficacy) [28,29]. This was attributed in part to the emergence of new variants, and it is possible that the drug is much less effective against Delta and subsequent variants. The MOVe-OUT trial began enrollment on 6 May 2021 and completed data collection on 4 November 2021. The FDA issued an emergency use authorization (EUA) on 23 December 2021. The EUA states that molnupiravir is not recommended for pregnant patients; however, it can be considered when these patients are at high risk of progressing to severe COVID-19 without other therapeutic options [11]. Molnupiravir has lower efficacy than the preferred treatment options. It is suspected to be effective against the BA.1 Omicron variant; however, in vitro and in vivo data are limited [26].

### 2.5. Nirmatrelvir+Ritonavir (Paxlovid)

Protease inhibitors nirmatrelvir [PF-07321332] and ritonavir are included within the oral antiviral paxlovid. Nirmatrelvir [PF-07321332] is a selective protease inhibitor of $M^{pro}$, also known as 3CL, a major enzyme necessary for SARS-CoV-2 replication [30]. PF-07321332 binds to 3CL through reversible thioimidate bond formation of Cys145 with a nitrile carbon. PF-07321332 is the antiviral portion of paxlovid and prevents replication, while ritonavir is a pharmacokinetic enhancer [31]. Ritonavir primarily inhibits cytochrome P450 enzymes, preventing the metabolism of protease inhibitors such as PF-07321332 [32,33]. Paxlovid contains nirmatrelvir [PF-07321332] and ritonavir in combination to ensure the highest efficacy of the antiviral effects.

Paxlovid has been approved for emergency use (EUA) on 22 December 2021, in high-risk adults and high-risk pediatric patients aged 12 and older with a minimum weight of 40 kg [34]. Paxlovid should be used to treat mild-to-moderate symptoms after a confirmed positive test result. Paxlovid should not be used in circumstances of pre-exposure or prevention. Refer to Table 1 for the FDA definition of high-risk categories. The dosing recommendations are 300 mg of nirmatrelvir with 100 mg of ritonavir twice daily for 5 days.

Extra precaution should be taken for those with a history of liver or kidney disease [35]. As paxlovid is renally cleared, dosing changes are recommended for those with eGFR ≥30 to <60 mL/min, with a decrease to 150 mg of nirmatrelvir with 100 mg of ritonavir, twice daily for 5 days [36]. Its use is not recommended in patients with severe renal impairment of eGFR <30 mL/min or severe hepatic impairment, as the use of paxlovid has not been studied enough in significant renal or hepatic dysfunction [36].

A double-blind clinical trial was conducted on non-hospitalized adults with specific high-risk factors with a confirmed positive COVID-19 test result. No patient had received a COVID-19 vaccine or had a history of infection. The results indicated that paxlovid reduced the risk of hospitalization or death by 89% if taken within 3 days of symptom onset [37].

There are still many concerns regarding the future direction of paxlovid, as well as many other approved outpatient therapeutics. One concern is the consideration from an ethical standpoint. The encouragement of the public to receive vaccinations is contrasted by only testing these therapeutics on unvaccinated people, leaving the potentially harmful effects of these regimens on vaccinated individuals still in question [38]. These concerns are also relevant to those with a history of COVID-19 infection, as paxlovid was also not studied in this population. Furthermore, there have been case reports of patients testing positive with COVID-19 again shortly after being treated with paxlovid; these patients would typically improve following treatment, with a recurrence of mild COVID-19 symptoms several days afterward, or would be asymptomatic with only a positive PCR test [39,40]. No known cases have progressed to severe COVID-19 as of yet, but further research needs to be conducted to better evaluate the frequency of recurrence and the implications for paxlovid therapy [41]. Lastly, while paxlovid is proven effective for the SARS-CoV-2 variants of concern, Delta and Omicron, studies have not yet confirmed paxlovid’s effects on subsequent Omicron sub-lineages [42]. Paxlovid is suspected to be effective against the B.1.1.519 and BA.2 Omicron sub-lineages [43] [Figure 1]. However, this remains in question as limited in vivo data and clinical efficacy are presented.

### 2.6. Evusheld

Evusheld is a combination of two monoclonal antibodies, tixagevimab and cilgavimab [44]. Tixagevimab and cilgavimab effectively work together to block the receptor binding protein of SARS-CoV-2 spike protein from binding the human ACE2 receptor, inhibiting viral attachment [45]. Thus, it is used as a pre-exposure prophylaxis, and is meant for those who are immunocompromised or immunosuppressed, who have not been recently exposed to an infected individual [46]. It is administered by injecting a 300 mg dose of tixagevimab and 300 mg of cilgavimab intramuscularly in non-exposed, immunocompromised individuals [46], as pre-exposure prophylaxis. Patients are tentatively recommended to receive injections at 6-month intervals, as the exact timing between dosing is not yet known [47]. According to the Phase III PROVENT trial, the relative risk reduction was 0.77, with a 95% CI of 0.46 to 0.90 [48]. The FDA issued Evusheld an EUA on 8 December 2021 and recently revised the EUA on 24 February 2022 [47,49]. Evusheld has shown to be efficacious against Omicron subvariants BA.1, BA.1.1 and BA.2 [50]. Only the Omicron BA.2 subvariant remains fully susceptible to Evusheld as BA.1 and BA.1.1 now have decreased susceptibility [51].

## 3. Inpatient Management

### 3.1. Remdesivir

As mentioned previously, remdesivir is an intravenous inhibitor of viral RNA-dependent RNA polymerase that is highly conserved across many coronaviruses, which makes remdesivir widely applicable as an antiviral agent [52], particularly in the SARS-CoV-2 virus. In the inpatient setting, remdesivir is recommended as a five-day total course of 200 mg IV on the first day, then 100 mg IV on each subsequent day prior to discharge, for a maximum of four additional days [23]. In the CATCO trial, remdesivir was associated with a small but significant reduction in progression to mechanical ventilation: 8.0% of patients on remdesivir required mechanical ventilation over the hospitalization, compared to 15% of patients randomized to receive standard of care at that time [53]. The SOLIDARITY trial showed a small but statistically significant mitigation of progression of disease and decreasing mortality in patients who were not ventilated; those that required ventilation showed no difference in being treated with either remdesivir or a placebo [54]. Interestingly, an earlier publication of the SOLIDARITY trial showed no difference in outcomes after administering remdesivir; this may be due to the smaller sample size of the earlier trial (2750 patients compared to a final count of 8275) or to a small clinical effect [55]. According to the ACCT trials, the number needed to treat for hospitalized patients was 26; the HR was 0.73 with a 95% CI of 0.52–1.03 [56]. For those with severe COVID-19, remdesivir is frequently used in conjunction with dexamethasone [57].

### 3.2. Corticosteroids

Of all the therapies studied thus far, corticosteroids have had the most unequivocal impact on mortality. The RECOVERY trial findings [58], released in July 2020, showed a significant reduction in 28-day mortality with dexamethasone compared to standard of care (age-adjusted rate ratio (aRR), 0.83; 95% confidence interval (CI), 0.75 to 0.93). Of note, there was a significant interaction with oxygen dependency. Among patients on mechanical ventilation (MV), the aRR was 0.64 (95% CI, 0.51 to 0.81), while, among those receiving supplemental oxygen without MV, the aRR was 0.82 (95% CI, 0.72 to 0.94). Additionally of importance, in patients not requiring oxygen supplementation, dexamethasone use, while not associated with a benefit, trended towards harm (aRR, 1.19; 95% CI, 0.91 to 1.55) [58]. In a large observational analysis from our New York City center of 1806 patients [59], we found similar results. Among patients with admission C-reactive protein (CRP) levels of ≥20 mg/dL, denoting a significant inflammatory burden, corticosteroid treatment was associated with a 75–80% reduction in the composite severe outcome of MV and mortality (adjusted odds ratio (aOR), 0.23; 95% CI, 0.08–0.70), while, among those with CRP ≤10 mg/dL, corticosteroid treatment was associated with severe COVID-19 outcomes (aOR, 2.64; 95% CI, 1.39–5.03). Several trial findings published later and analyzed in a WHO meta-analysis [60] have reinforced the findings that corticosteroids have a mortality benefit in the critically ill patients with COVID-19, as defined in Table 2.

While great progress has been made in utilizing corticosteroids for COVID-19 treatment, several clinically relevant questions warrant further research and are discussed below.

### 3.3. Heterogeneity of Response across the Clinical Severity Spectrum

In the RECOVERY trial, patients requiring supplemental oxygen but not on MV included those who received both low and high oxygen supplementation [58]. While this subgroup overall benefited from corticosteroids, the differences in response based on a low versus high level of oxygen requirement were not established. Given the risk of harm in patients with milder disease, further stratifying this subgroup for granular assessment of response to corticosteroids among those requiring low oxygen supplementation is clinically relevant.

In addition, inflammatory biomarkers could also play an important role in risk stratification. Patients with a low oxygen requirement but high inflammatory burden may represent a subgroup at risk for progressing to a critical disease state and could be more likely to benefit from corticosteroids than patients with a low oxygen requirement and low inflammatory burden or even no oxygen requirement and high inflammatory burden. Further studies to prognosticate based on clinical variables will be informative.

### 3.4. Impact on Long-Term Autoreactivity

Recent studies have demonstrated heightened autoreactivity in patients with severe COVID-19 [61,62,63]. Patients with a higher inflammatory burden, based on elevated CRP, are likely to test positive for antinuclear antigen (ANA) and rheumatoid factor (RF) [64]. In an elegant study, using Rapid Extracellular Antigen Profiling (REAP), Wang et al. [63] have demonstrated a diffuse array of autoantibodies directed against cytokines and chemokines. While the functional effect of these antibodies remains unclear, early data suggest that they may directly neutralize the activity of cytokines/chemokines and alter immune function in COVID-19 patients [63]. Increased autoreactivity seems to correlate with severe disease [63]. Whether this is a direct effect of pathogenic antibodies or an uncontrolled response to the persistence of antigens is unclear. Patients with demonstrable antibodies to interferon-α had a persistently higher viral load compared to antibody-negative controls, suggesting impaired clearance due to an impaired interferon-α-mediated viral clearance pathway [65]. Whether these antibodies cause tissue-specific damage and are associated with persistent symptoms as seen in “long-COVID” patients remain unclear.

Corticosteroids are well-known inhibitors of cytokines and chemokines, and effective in reducing inflammation and autoantibody production. This inhibition has to be balanced against the deleterious effect of inhibiting interferon-α-mediated viral clearance. It may be possible that corticosteroids are most effective in patients who have demonstrable increased autoreactivity, and further research should test this hypothesis.

### 3.5. Predictors of Early Response

In a recent observational study of 2707 patients, of whom 324 received corticosteroids, a CRP response, defined as a ≥ 50% reduction from admission value within 72 h, was associated with a significant reduction in mortality compared to CRP non-response (adjusted OR 0.27; 95% CI 0.14, 0.54) [45,64]. This suggests that CRP may be a biomarker to predict the early response to corticosteroids.

Other clinical variables and biomarkers that could predict early response are of great interest. Candidates include the neutrophil lymphocyte ratio (NLR), neutrophil monocyte ratio (NMR) and d-dimer. COVID-19 is associated with lymphocyte and monocyte recruitment to the lungs, the primary site of injury, facilitated by cytokines such as interleukin (IL-6) and monocyte chemoattractant protein-1 (MCP-1). Corticosteroid-treated patients may show improvements in lymphocyte counts, monocyte counts and perhaps d-dimer.

### 3.6. Reactivation of Latent Infections

The impact of corticosteroids on infections, both new and reactivated, is an important consideration. Strongyloides hyperinfection and reactivation due to corticosteroid therapy is well established [66,67]. Disseminated Strongyloidiasis, associated with high mortality, can occur with corticosteroids, other immunomodulatory agents and hematologic malignancies [66]. Such cases have been reported even with low-dose and short-duration corticosteroid therapy (3 mg dexamethasone equivalent and duration 5 days) [67]. Empiric prophylactic therapy with ivermectin in patients in endemic areas, or more broadly in countries other than Australia, North America or Western Europe, may be a reasonable strategy. Disseminated Strongyloidiasis should be considered as a differential in COVID-19 patients on corticosteroids with unexplained Gram-negative bacteremia and acute clinical decompensation [68].

Other latent infections of concern include tuberculosis, hepatitis B and herpes. Dexamethasone stimulates the reactivation of HSV-1 ex vivo [69,70] and in animal studies, and it may reactivate the closely related bovine herpesvirus 1 (BHV-1) in latently infected calves [71]. There are little data on the reactivation of hepatitis B and tuberculosis with short-term steroid use.

Corticosteroids are one of the few therapies with an unequivocal benefit in COVID-19, including a mortality benefit in the subgroup of severely ill patients. They are inexpensive and available universally, including in regions with limited resources. However, it is important to take into account the potential for harm due to corticosteroids. Biomarkers such as CRP may help to stratify patients who are more likely to benefit and can also serve as an early therapeutic response biomarker. Patients on corticosteroids should be monitored for the reactivation of Strongyloides and prophylactic ivermectin should be considered in patients from highly endemic areas.

### 3.7. Baricitinib

Baricitinib belongs to a class of medications called Janus kinase inhibitors, or JAK inhibitors. These medications act by inhibiting signal transducer and activator of transcription proteins, also known as STAT proteins. STAT proteins play integral roles in cellular replication, regulating processes such as growth, replication, signaling and apoptosis [72]. JAK inhibitors are frequently used in oncologic settings, in order to attempt to control rapidly dividing cancer cells. By the same token, JAK inhibitors were trialed to treat COVID-19 with the rationale that they might be able to inhibit the overactivation of the immune system [73]. Interestingly, of the JAK inhibitors, only baricitinib and tofacitinib have been shown to have efficacy in treating COVID-19. In the ACTT-2 trial, baricitinib with remdesivir was shown to increase the recovery rate by a day (7 days compared to 8 days) when compared to remdesivir alone [74]; the study also showed a small improvement in outcomes overall at day 15, though it was not statistically significant. A subsequent study, the COV-BARRIER trial, also established the benefit of baricitinib when used in conjunction with standard of care, most notably corticosteroids. The COV-BARRIER trial showed that although bariciticib did not impact the overall progression of the disease, defined as increasing oxygen requirements including mechanical ventilation, it did improve all-cause mortality at 28 days, with a low number needed to treat of 20 patients [75]. The primary limitation of baricitinib is renal dysfunction, and it is explicitly not recommended to be used in patients with eGFR < 15. The recommended dosing is based on renal clearance (4mg daily for those with eGFR > 60, 2mg daily for those with eGFR 30–60, 1mg daily for eGFR 15–30), and the treatment duration is up to 14 days or until hospital discharge. Patients most likely to benefit from baricitinib are those with high oxygen requirements, defined as BiPAP or HFNC, with an unclear though possible benefit in those patients requiring mechanical ventilation [75].

### 3.8. Tocilizumab

Tocilizumab is a monoclonal antibody instructed for use in hospitalized patients with both mild/moderate and severe COVID-19 symptoms. Tocilizumab is effective in treating COVID-19-induced cytokine storms since it is an IL-6 receptor antagonist [76]. The advised use of tocilizumab consists of injecting 8 mg per kg of patient body weight as a single IV dose [56]. It has been shown to be highly effective in hospitalized COVID-19 patients presenting with hypoxia with oxygen saturation of <92% and elevated markers of systemic inflammation, most notably CRP ≥ 75 mg/L, when administered in addition to dexamethasone [65]. According to the RECOVERY clinical trial, the number needed to treat was 33, with a risk ratio of 0.85 and 95% CI of 0.76 to 0.94 [25,77]. This trial began enrollment on 23 April 2020 and ended on 24 January 2021. Limitations of this treatment include the use of tocilizumab in combination with baricitinib due to increased risk of infection from potent immunosuppressors. The FDA issued an EUA on 24 June 2021, for tocilizumab use.

### 3.9. Anakinra

During a COVID-19 infection, many inflammatory markers are increased, including interleukin-1. Anakinra is a recombinant IL-1 receptor antagonist, most commonly used in the treatment of rheumatoid arthritis and cryopyrin-associated periodic syndromes [78]. In the SAVE-MORE trial, treatment with anakinra yielded improved outcomes for patients with hypoxia requiring supplemental oxygen and a suPAR biomarker at a serum concentration of ≥6 ng/mL [79]. Specifically, the incidence of severe respiratory failure was decreased from 59.2% in standard of care to 22.3% in those treated with anakinra, with a 10.8% improvement in 30-day mortality as well when compared to standard of care [79]. Despite these promising results, other studies, including REMAP-CAP and CORIMUNO-ANA-1, found no benefit for the use of anakinra in patients with COVID-19 at large [80,81]. Thus, there is an apparent importance of risk stratification with suPAR, which is an assay that is not readily available in many countries, including the United States. As a result, there is no recommendation for the use of anakinra in the United States, either in favor or against. In Europe, anakinra is approved for use in patients with COVID-19 who require supplemental oxygen with a suPAR level of ≥6 ng/mL, at a dose of 100 mg as a subcutaneous injection for 10 days [82]. Anakinra is expected to be effective against the Omicron variant, though there are no known active studies investigating this specifically [83].

### 3.10. Anticoagulation

Heparin is an anticoagulant utilized for treatment in hospitalized patients with mild/moderate and severe COVID-19 symptoms. While the specific mechanism of heparin’s action is unknown, there is great evidence for low-molecular-weight heparin exhibiting anti-inflammatory and anti-viral benefits in patients with severe SARS-CoV-2 [84]. It is advised to use heparin in different manners depending on the therapeutic or prophylactic dose usage. The NIH panel recommends administering a prophylactic dose of heparin in non-pregnant, hospitalized patients requiring mechanical ventilation [56]. A therapeutic dose is preferred in patients who have moderate disease, defined as having symptomatic COVID-19 disease but not requiring mechanical ventilation, HFNC, CPAP, BiPAP or pressor support and with no contraindications to anticoagulation, such as platelets <50 × 10^9^/L, hemoglobin <8 g/dL, being on dual antiplatelet therapy or having had major bleeding within the past month [14]. According to the RAPID trial, the number needed to treat was 8, with an indicated relative risk of 0.68 and with a 95% CI of 0.49 to 0.96 [85]. The FDA issued an abbreviated new drug application (ANDA) approval for heparin in relation to COVID-19 treatment on 15 July 2020. 

**Figure 2:**
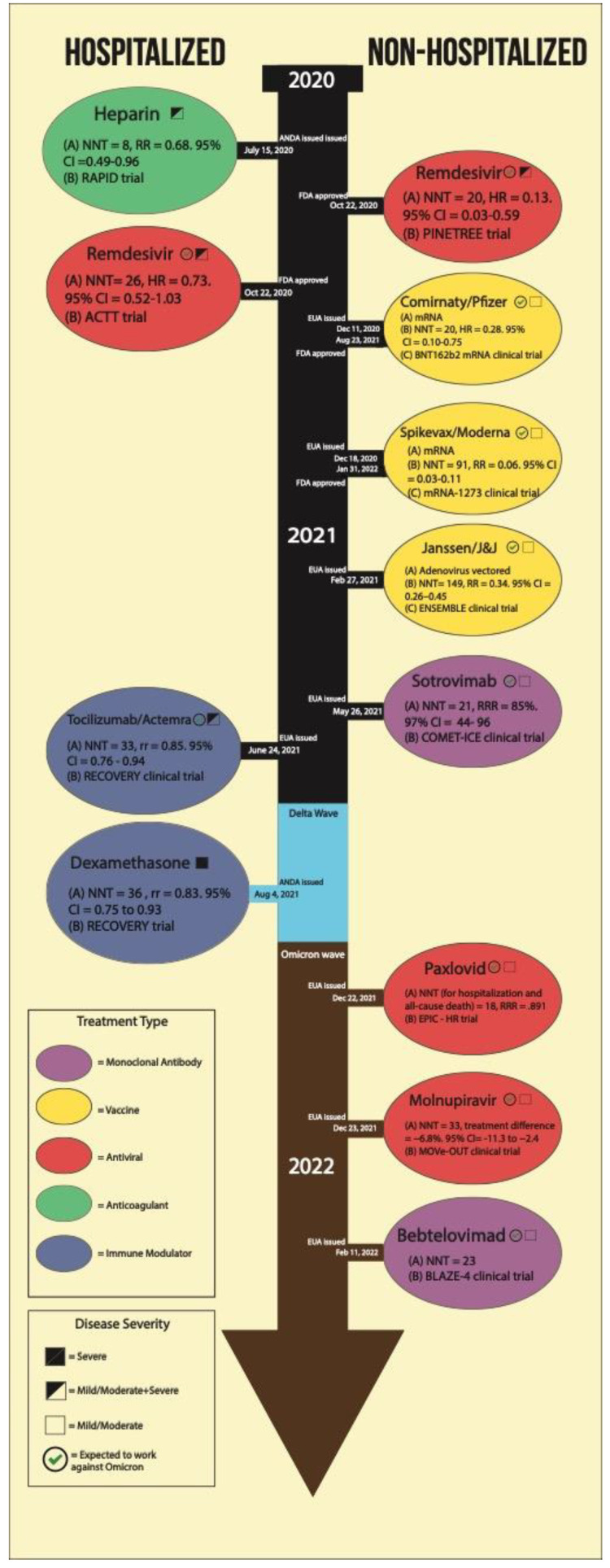
Timeline of COVID-19 therapeutics and authorization use issuance at the FDA.

A timeline of FDA approvals for each drug mentioned in this review can be seen in Figure 2. 

## 4. Conclusions

In this rapidly evolving landscape, it is imperative to stay abreast of current therapeutics and their efficacy, particularly against newer and rapidly changing strains of COVID-19. In this brief yet comprehensive review, we discuss the therapeutics available for the treatment of COVID-19 infection that are shown to be effective in well-designed randomized controlled trials. It is worth noting the rapid speed with which many of these therapeutics were identified and developed, which is a testament to the massive undertaking that many international consortia performed, including platform trials such as RECOVERY (Randomised Evaluation of COVID-19 Therapy), REMAP-CAP (A Randomised, Embedded, Multi-factorial, Adaptive Platform Trial for Community-Acquired Pneumonia), ACTIV-IV (Accelerating COVID-19 Therapeutic Interventions and Vaccines) and ATTACC (Antithrombotic Therapy to Ameliorate Complications of COVID-19). It cannot be overstated how much progress has been made in these last two years, and how far we have come from March 2020, when our only interventions were a trial of hydroxychloroquine, a ventilator and a strong dash of hope.

Finally, though beyond the purview of our article, vaccines against the SARS-CoV-2 virus still remain the mainstay of saving lives, and their importance as the most effective preventative measure cannot be emphasized enough. Nevertheless, our aim with this article is to educate providers of the breadth of therapeutics available in both inpatient and outpatient settings, tailored to disease severity. In doing so, we hope to facilitate the selection of the most appropriate agent in each clinical setting and continue to improve outcomes in the treatment of COVID-19.

## Figures and Tables

**Table 1:** Risk factors for progression to severe disease based on FDA and NIH recommendations.

1. Aged ≥ 65 years
2. Obesity (BMI > 30)
3. Diabetes mellitus type 2
4. History of CAD, hypertension, congenital heart disease
5. History of respiratory disease, such as COPD, moderate or severe persistent asthma, interstitial lung disease, cystic fibrosis, pulmonary hypertension
6. Sickle cell disease
7. Immunosuppressive regimen
8. History of: cancer, chronic liver disease, chronic lung diseases, dementia or other neurological conditions, diabetes, Down syndrome, HIV infection, Immunocompromised, mental health conditions: depression, schizophrenia, sickle cell disease, tuberculosis, substance use disorders, stroke or cerebrovascular disease, organ or blood stem cell transplant
9. Chronic kidney disease
10. Are overweight, obese, pregnant, smoke [14].

NOTE: FDA = Food and Drug Administration; NIH = National Institutes of Health; BMI = Body Mass Index; CAD = Coronary Artery Disease; COPD = Chronic Obstructive Pulmonary Disease; HIV = Human Immunodeficiency Virus.

**Table 2:** Summarizing the indications for use of corticosteroids in COVID-19.

**Corticosteroids are beneficial**
1. Moderate to severe ARDS (defined using Berlin Criteria) and need for invasive mechanical ventilation
2. Moderate to severe ARDS requiring non-invasive mechanical ventilation (high flow nasal cannula)
3. Mild ARDS (pao2/fio2 < 300) and requiring oxygen support
4. Pneumonia severity index (PSI) > 130
**Corticosteroids may be beneficial**
1. ARDS and elevated inflammatory markers (CRP > 20 mg/dL)
**Corticosteroids may be harmful**
1. Mild to moderate disease not requiring oxygen support
2. Mild to moderate disease and low inflammatory markers (CRP < 20 mg/dL)

NOTE: ARDS = Acute Respiratory Distress Syndrome; CRP = C-reactive Protein.

## Data Availability

Not applicable.
